# Impact Resistance of Styrene–Butadiene Rubber (SBR) Latex-Modified Fiber-Reinforced Concrete: The Role of Aggregate Size

**DOI:** 10.3390/ma15041283

**Published:** 2022-02-09

**Authors:** Muhammad Rehan Ashraf, Usman Akmal, Nauman Khurram, Fahid Aslam, Ahmed F. Deifalla

**Affiliations:** 1 Department of Civil Engineering, University of Engineering and Technology Lahore, Punjab 54890, Pakistan; rehanashraf@uet.edu.pk (M.R.A.); usman.akmal@uet.edu.pk (U.A.); nauman@uet.edu.pk (N.K.); 2 Department of Civil Engineering, College of Engineering in Al-Kharj, Prince Sattam bin Abdulaziz University, Al-Kharj 11942, Saudi Arabia; 3 Department of Structural Engineering and Construction Management, Faculty of Engineering, Future University in Egypt, 90th Street, New Cairo 11835, Egypt; ahmed.deifalla@fue.edu.eg

**Keywords:** impact resistance, polypropylene fibers, SBR latex, aggregate size effect, drop weight

## Abstract

Improvements in tensile strength and impact resistance of concrete are among the most researched issues in the construction industry. The present study aims to improve the properties of concrete against impact loadings. For this purpose, energy-absorbing materials are used along with fibers that help in controlling the crack opening. A polymer-based energy-absorbing admixture, SBR latex, along with polypropylene fibers are used in this study to improve the impact resistance. Along with fibers and polymers, the effect of the size of aggregates was also investigated. In total, 12 mixes were prepared and tested against the drop weight test and the Charpy impact test. Other than this, mechanical characterization was also carried out for all the 12 concrete mixes. Three dosages of SBR latex, i.e., 0%, 4%, and 8% by weight of cement, were used along with three aggregates sizes, 19 mm down, 10 mm down, and 4.75 mm down. The quantity of polypropylene fibers was kept equal to 0.5% in all mixes. In addition to these, three control samples were also prepared for comparison. The mix design was performed to achieve a normal-strength concrete. For this purpose, a concrete mix of 1:1.5:3 was used with a water to a cement ratio of 0.4 to achieve a normal-strength concrete. The experimental study concluded that the addition of SBR latex improves the impact resistance of concrete. Furthermore, an increase in impact resistance was also observed for a larger aggregate size. The use of fibers and SBR latex is encouraged due to their positive results and the fact that they provide an economical solution for catering to impact strains. The study concludes that 4% SBR latex and 0.5% fibers with a larger aggregate size improve the resistance against impact loads.

## 1. Introduction

Concrete is widely used in various civilian and military buildings in Pakistan and is one of the most important construction materials used all over the world. The main concern related to concrete is its low tensile strength and weak resistance against cracking, which makes it brittle. Terrorism is the biggest problem of many countries on the globe these days. Among these countries, Pakistan has witnessed many major terrorism attacks on important public and private sector buildings in the recent past, which inflicted many deaths and left hundreds with wounds. It has been observed that the major cause of loss of life was the severe damage of structural elements during terrorist attacks resulting in the failure of the building. Furthermore, investigations have highlighted that the conventional concrete used in those structures subjected to blast loadings was not resistant enough to bear such high strain loadings [1,2].

Among different important mechanical properties of concrete, impact resistance is the one which needs to be carefully studied to make the reinforced concrete (RC) structure blast-resistant. In this regard, researchers are carrying out extensive research on the improvement in the impact resistance of normal-strength concrete. To improve the impact resistance of concrete, various advanced materials, such as man-made and natural fibers, have been extensively studied in various experiments [3,4,5]. The use of ultra-high-performance concrete is one of the options to make the structure blast-resistant, but generally, this type of concrete is very expensive. Some advanced composite materials, such as carbon fiber-reinforced polymers, have also been employed in such applications, which is also a very costly material [6]. The use of costly materials is, most of the time, not acceptable, considering the impact on the overall cost of the project. Therefore, exploring alternate cost-effective materials to improve the impact resistance of normal concrete is the need of the day and this work is the way forward.

Xu et al. [7] studied the mechanical performance of fiber- and polymer-modified concrete by using polyester fibers and SBR latex. They found that strength properties, impact, and flexural toughness significantly improved. They investigated that SBR latex produced a continuous film of polymer inside the concrete, which enhanced the compactness and toughness of ITZ by retaining the water for hydration. They also concluded through microscopic test that SBR latex acts at a retarder at early ages of hydration (within 3 days). However, this effect was no more observed in later age (28 day later).

Wang et al. [8] revealed an increase in toughness, flexural strength, and tensile bond strength by the allowance of styrene–butadiene rubber (SBR) latex. They also concluded that compressive strength and flexural strength are directly proportional to the bulk density when the polymer to cement ratio is kept below 10% but it has an adverse effect on the mechanical properties of the polymer to cement ratio, which increased above 10%. This indicates that a higher proportion of polymer admixture is not beneficial for the mechanical properties of concrete.

Another similar study [7] concluded that the inclusion of fibers along with SBR latex improves the impact resistance of concrete. The most optimal concentration of SBR latex is 90 kg/m^3^, which comes out to be around 4% of the cement weight. The results obtained in the research also concluded that the addition of SBR latex has no significant effect on cement hydration in the long term. Besides, continuous SBR latex films formation presented in cement substrates makes it possible to raise toughness and compact degree of the interface transition zone (ITZ). Furthermore, SBR latex triggers fiber and cement paste, which then leads to a tight mutual connection.

Along with polymers, various types of fibers have also been studied to improve the impact resistance of ordinary concrete. Fibers are mainly categorized into two types, namely flexible fibers and stiff fibers. Polypropylene and carpet nylon are some typical examples of flexible fibers, while steel fibers, basalt fibers, and carbon fibers are classified as stiff fibers.

Fu et al. [9] studied the impact response of concrete reinforced with fibers and concluded that flexible fibers (e.g., polypropylene) can improve the crack width and impact resistance of concrete due to their high ductility, whereas stiff fibers contribute partly to the strength of concrete. With the increasing strain rate, mechanical properties and energy absorption capacity of concrete also increase. Due to the addition of fibers, mechanical properties, as well as impact resistance properties, improve especially under high-strain-rate impact loading, such as in the drop weight test. In a similar study, Hosseini et al. [10] and Yin et al. [11] utilized recycled polypropylene carpet fibers to improve impact resistance and energy absorption capacity. It was concluded that the recycled PP fibers significantly improved impact load from 71 to 418% for a fiber volume of 0.25–1.25%. However, some studies suggest that the optimum dosage of PP fibers should be kept between 0.25 and 0.5% to improve the compressive and flexural strengths [12]. Some researchers [13] have also studied the combined effect of pp fiber along with SBR latex and concluded that both SBR latex and polypropylene fibers improve the impact toughness of concrete due to higher energy absorption characteristics of SBR and bridging action contributed by fibers. The effect of fibers in absorbing the impact load and cracks was observed in post-crack toughness of polymer-modified concrete with an increase in the dosage of fibers from 0.1% to 0.3%.

Besides the addition of fibers and polymers, many researchers have also investigated the effect of aggregate size on the impact resistance of concrete. Most studies concluded that for a normal-strength concrete, both strength and fracture energy increase with the increase in coarse aggregate size [14,15,16]. However, some studies reflect the mixed view of the effect of aggregate size on fracture energy. According to [17], the size of coarse aggregate has a direct influence on concrete strength but the fracture energy of concrete decreases when the aggregate size is increased. Karamloo et al. [18] studied the effects of maximum aggregate size on fracture behavior of self-compacting lightweight concrete. It was concluded that when the size of aggregate is increased from 9.5 mm to 19 mm, it increases the fracture energy by 47.7% for the water to cement ratio of 0.35 and 86.7% for the water to cement ratio of 0.4%. It was also reported that for the same water to cement ratio, the fracture energy decreased for a larger aggregate size.

ACI 544 [19] specifies the most common type of test to measure the impact resistance of concrete. In this study, a drop weight test is used to evaluate the resistance of concrete against impact loading. This is a simple and economical test. However, the results of this test cannot be relied on fully due to the non-homogeneous nature of concrete. In this regard, Zhu et al. [20] used a U-shape specimen to measure the resistance of concrete against impact loading. The advantage of this specimen shape is that the location and propagation of the crack are predetermined. The test results of U-shaped specimens depicted the maximum coefficient of variation value equal to 31.2%, which was lower than the values of maximum coefficient of variation obtained from the traditional disc specimens as specified by ACI-544 [19].

The literature review revealed that the addition of fibers and the use of polymers in concrete is an effective approach to enhance its impact resistance. In most previous research studies, the combined effect of fibers and polymer has been studied on the impact resistance of concrete. Many studies have also investigated the effect of aggregate size variation on the fracture energy and compressive strength of concrete. However, the influence of aggregate size along with fibers and polymers on the impact resistance of concrete is rarely investigated. Thus, the present study is focused to explore the combined effect of polypropylene fiber and SBR latex along with the variation of aggregates size. Further, the suitability of a non-ACI test specimen, i.e., U-shapes, was also investigated.

## 2. Experimental Program

### 2.1. Materials

#### 2.1.1. Cement and Aggregates

Ordinary Portland cement (OPC) of type-1, complying with ASTM C-150 [21] and having a specific gravity of 3.1 kg/m^3^, was used in all concrete mixes. Locally available river sand (Lawrencepur) and crush stone (Sarghodha) were used as fine and coarse aggregates, respectively. For coarse aggregates, three different maximum aggregate sizes, i.e., 4.75 mm, 10 mm, and 19 mm, were used. Properties of fine and coarse aggregates are given in Table 1.

#### 2.1.2. SBR Latex

**Figure 1:**
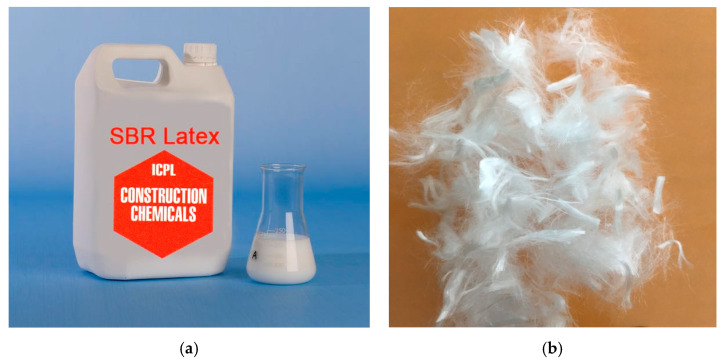
(**a**) SBR latex and (**b**) polypropylene fibers.

Polymer admixture, known as SBR latex (styrene–butadiene rubber), is usually considered as a binding agent. Further, the formation of polymer structure within the concrete matrix also imparts a positive effect on the impact resistance of concrete [1]. Different quantities of SBR latex have been investigated in the past, as indicated by literature review. In the present study, the dosage of SBR latex, conforming to ASTM C1042 [22] as 4% and 8% of cement weight, has been used in line with the recommendation of the literature and guidelines of the manufacturer. Properties of SBR latex are given in Table 2 and a picture is presented in Figure 1a.

#### 2.1.3. Polypropylene Fibers

It is a well-established fact that fibers of different types improve the mechanical properties (flexural strength, tensile strength, impact resistance, etc.) of plain concrete, particularly in post-crack regions [11]. In this present study, the effect of adding polypropylene fibers in concrete, with and without SBR latex at a constant dosage of 0.5% by weight of cement, was investigated [12]. The polypropylene fibers had a maximum length of 12 mm, as shown in Figure 1, and their characteristics are mentioned in Table 3. Fibers were sprinkled manually during the dry mixing of concrete in the mixer.

### 2.2. Concrete Mixes

A total of 12 concrete mixes, using (were prepared) 3 different coarse aggregate sizes and 2 distinct SBR latex at dosages, were prepared. In all mixes, polypropylene fibers were included at constant dosage (0.5%), except the control mixes. Among twelve concrete mixes, three were control mixes, which were prepared without the use of SBR latex and fibers, and three mixes contained only polypropylene fibers. The remaining six concrete mixes were prepared using both polypropylene fibers and SBR latex with specific aggregate sizes. Each concrete mix is referred to with a proper designation as mentioned in Table 4. In a typical mix 4.75-F-4S, 4.75 is an aggregate size in millimeter, while F represents the presence of fibers and 4S stands for 4% dosage of SBR latex. Similarly, C-4.75 indicates a control concrete mix with 4.75 mm without fibers and SBR latex, and 4.75-F represents a concrete mix with 4.75 mm aggregates with fibers only. To design a normal-strength concrete, a mix ratio of 1:1.5:3 of concrete ingredients was used with well-graded aggregate by sticking with the maximum size of the aggregate of each category. The water to cement ratio was kept equal to 0.4 in each case. The concrete mix ingredients per cubic meter for different concrete mixes are presented in Table 4. The slump value of all mixes varied from 20 mm to 40 mm, depending on the SBR latex dosage.

### 2.3. Detail of Specimens

The following concrete specimens were cast to determine the mechanical properties and impact resistance of concrete mixes. Details of the specimen are provided in Table 5.

**Figure 2:**
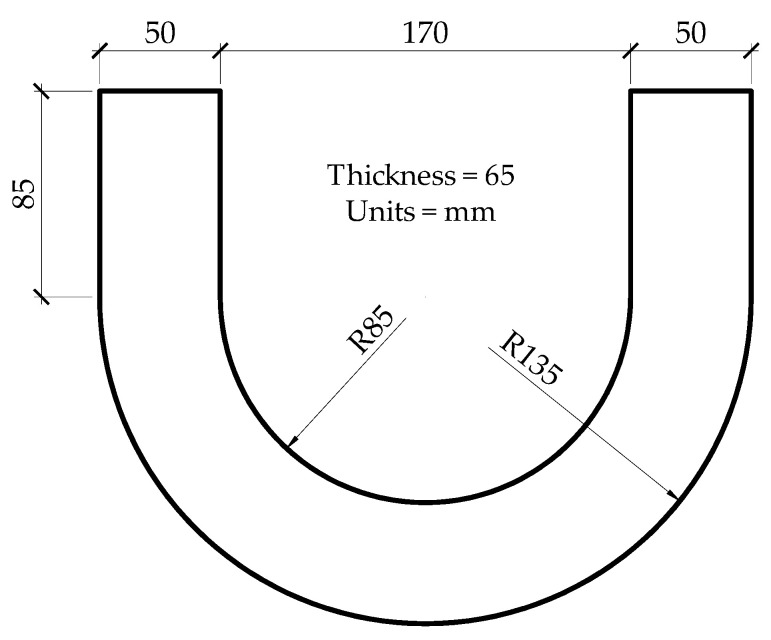
U-Shape specimen for the drop weight test.

**Figure 3:**
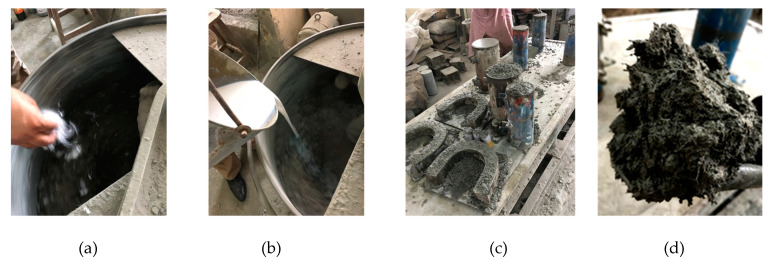
Various steps involved in concrete mixing: (**a**) addition of polypropylene fibers during dry mixing; (**b**) inclusion of liquid-based SBR latex; (**c**) vibration after casting; and (**d**) fibrous concrete after mixing.

(1) Concrete cylinders of standard size of 150*ϕ* × 300 mm were used for determining mechanical properties, which include compressive strength, MOE, and split cylinder tensile strength.
(2) Concrete cylinders of size of 40*ϕ* × 300 mmwere usedfor preparing disc specimens for the Charpy impact test.
(3) The concrete specimens for the drop weight impact test are as follows:

Concrete cylinders with a size of 100*ϕ* × 200 mm were used for preparing cylindrical discs of 63.5 mm height for drop weight test, as specified by ACI 544 [19];

U-shape specimens, according to the dimensions suggested by Zhu et al. [20], as shown in Figure 2. For this purpose, special molds were prepared in the laboratory as shown in Figure 3.

### 2.4. Preparation of Test Specimens

Required quantities of all ingredients for a particular concrete mix were first used to dry mixes, along with the addition of fibers through manual sprinkling. This dry mixing process was continued until a uniform mix was achieved. The average time for this process was observed to be 4 to 5 min for each concrete mix. After the addition of water along with a required quantity of SBR latex, the wet mixing was continued for another three minutes. Before pouring concrete into molds for slump test, all steel molds were oiled before pouring concrete. The compaction of concrete was carried out through the vibrating table. The specimens were demolded after 24 h of casting, and then concrete specimens were placed in the curing room for 28 days. Figure 3 shows the various steps involved in the concrete mixing.

**Figure 4:**
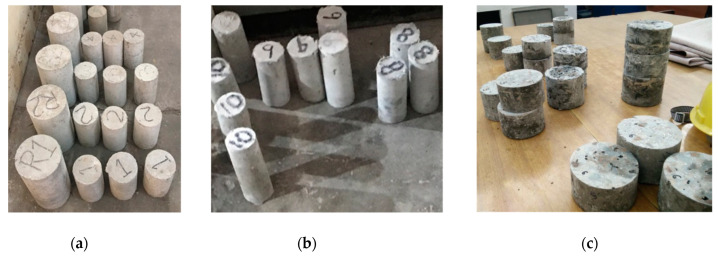
Hardened samples after 28 days curing (**a**) cylinder for compression strength and split cylinder test, (**b**) samples for the Charpy impact test, (**c**) cylindrical disks for the drop weight test.

The compression and modulus of elasticity tests were performed on the cylindrical samples after 28 days. The test specimens used for drop weight were prepared by cutting the cylinders (100 mm in diameter × 200 mm in length) to obtain the disc height of 63.5 mm from the central region of the concrete cylinder. This method of obtaining cylindrical disc specimens is based on the ACI 544 [19]. Figure 4 shows the hardened concrete specimens.

### 2.5. Testing Procedure

For the mechanical characterization of SBR-latex-modified fiber-reinforced concrete, various tests were performed in the laboratory.

#### 2.5.1. Mechanical Tests

**Figure 5:**
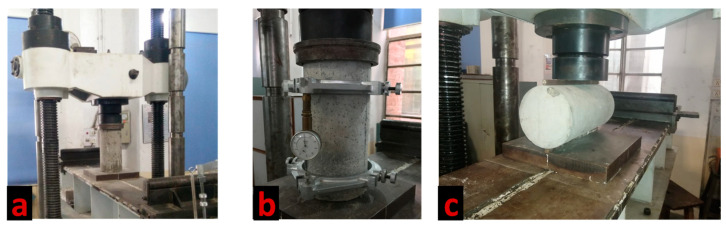
Experimental setup for mechanical testing (**a**) compressive strength test, (**b**) modulus of elasticity test, (**c**) split cylinder strength test setup.

For mechanical characterization, all the testing was conducted in a fully calibrated universal testing machine, following a proper ASTM procedure, i.e., ASTM C39 [23], ASTM C469 [24], and ASTM C496 [25] were used to determine the compression strength, the modulus of elasticity (MOE), and the split cylinder tensile strength, respectively. Figure 5 illustrates the specimen in UTM during testing.

#### 2.5.2. Impact Tests

In this study, two types of impact tests were performed. Impact resistance tests were conducted in accordance with ACI 544.2R-89 [19].

(a) Drop weight impact test

**Figure 6:**
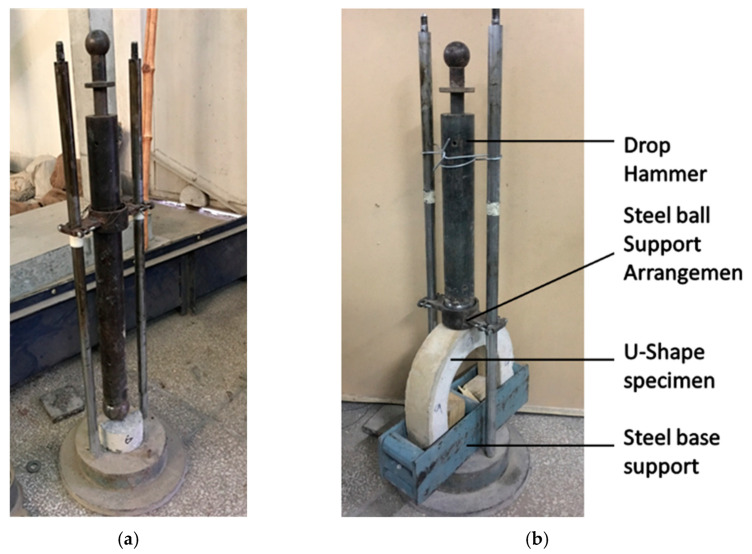
Arrangement for drop weight (**a**) drop weight test, (**b**) U-shaped samples.

The arrangement for the drop weight impact test was prepared in the lab, as shown in Figure 6a. A hammer weighing 4.53 kg (10 lbs) was used with a drop height of 457 mm for applying the impact load on a cylindrical disc which was 100 mm in diameter and 63.5 mm in height. For this test, the hammer was dropped on a steel ball and, through this steel ball, the impact load was transferred to a cylindrical disc.

In the case of the impact load test on the U-shape specimen, a 2.5 kg (5.5 lbs) hammer was used with a drop height of 128 mm. In this test, the U-shape specimen was fixed at the bottom of the testing setup at the required position with respect to the line of action of load, as shown in Figure 6b. It may be noted here that there is a gap between the ends of the specimen and testing setup, which is required to allow free lateral movement of the specimen during impact loading. It is pertinent to mention here that the impact load causes bending in the U-shape specimen. The impact energy in the drop weight tests, corresponding to initial cracking and final failure, was calculated using Equations (1) and (2).

(1) $$N_{1}=B_{1}\times mgh$$

(2) $$N_{2}=B_{2}\times mgh$$

where

*N*_1_ = the drop weight potential energy at initial cracking;
*N*_2_ = the drop weight potential energy at final failure;
*B*_1_ = the number of blows at initial cracking;
*B*_2_ = the number of blows at final failure.

(b) The Charpy impact test

**Figure 7:**
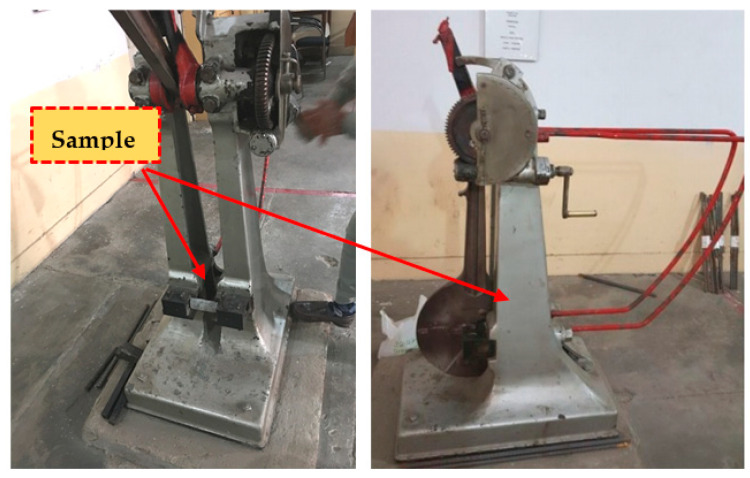
Test specimen in the Charpy impact machine.

During the Charpy impact test, a flexural impact loading was induced by a pendulum fork, as shown in Figure 7. The impact energy requires the sample to fail to compute the difference in energies before (free fall energy) and after the breaking of the specimen. The energy was measured in terms of the angle attained by the fork before and after striking, and then a difference of energies was computed by the expression given in Equation (3). This test gives an idea about the type of material, as a ductile material will absorb more energy before failing and a brittle material will absorb less energy before failure. Figure 7 shows the test specimen placed in the Charpy impact test machine.

(3) $$\mathrm{Impact}\;\mathrm{Energy},\;\Delta E=mgR(\cos\theta_{2}-\cos\theta_{1})$$

where

M = the mass of fork = 22.9 kg;
*R* = the radius of fork = 700 mm;
*θ*_1_ = the free-fall angle;
*θ*_2_ = the post-impact angle.

## 3. Results and Discussion

### 3.1. Compressive Strength and Modulus of Elasticity

**Figure 8:**
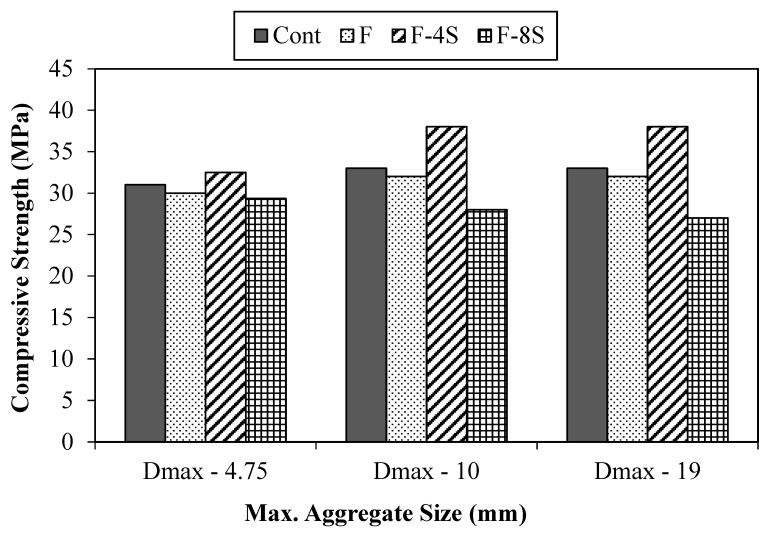
Compressive strength results.

The results of compressive strength values for all 12 concrete mixes are arranged in three groups with reference to the size of the maximum aggregate used in the study, as shown in Figure 8. It is pertinent to mention here that the results presented are an average of three samples of each concrete mix. The compressive strength results indicate that all concrete mixes attained the value of compressive strength, ranging from 27 MPa to 38 MPa, which can be considered as normal-strength concrete. There were three groups of concretes with different aggregate sizes.

A similar trend of compressive strengths values was found in all of these three groups. When fiber was added to concrete, the strength of the fibrous mixes was slightly dropped, and this observation is in line with the previous research studies on fiber-reinforced concrete. The reason for this drop in compressive strength is the reduced density of concrete mix due to the addition of fibers. It is evident from the results that when 4% SBR latex was added, the compressive strength of the resulting concrete mix slightly improved. The maximum improvement of 15% was observed for the case of the F-4S group (0.5% fibers and 4% SBR latex) as capered to the control mix. This was true for both concrete mixes made using the aggregate with maximum sizes of 10 mm and 19 mm. The improvement in compressive strength due to the addition of SBR latex is mainly due to the formation of polymer structure, which results in an enhancement of concrete density. However, when 8% SBR latex was added, the compressive strength of the resulting concrete mix reduced, far less than that of the compressive strength of the control mix. The maximum drop of 18% (33 MPa to 27 MPa) was exhibited by concrete mix made using the maximum aggregate size of 19 mm (i.e., 19-F-8S) when compared to the corresponding control mix. A change in the hydration kinetics due to excessive quantity of SBR latex or internal cracking of concrete matrix, as a result of excessive formation of polymer structures, could explain the detrimental effects of 8% SBR latex on compressive strength. Therefore, it is recommended that the microstructure of such concrete mixes must be studied to highlight the reason for the drop in compressive strength.

**Figure 9:**
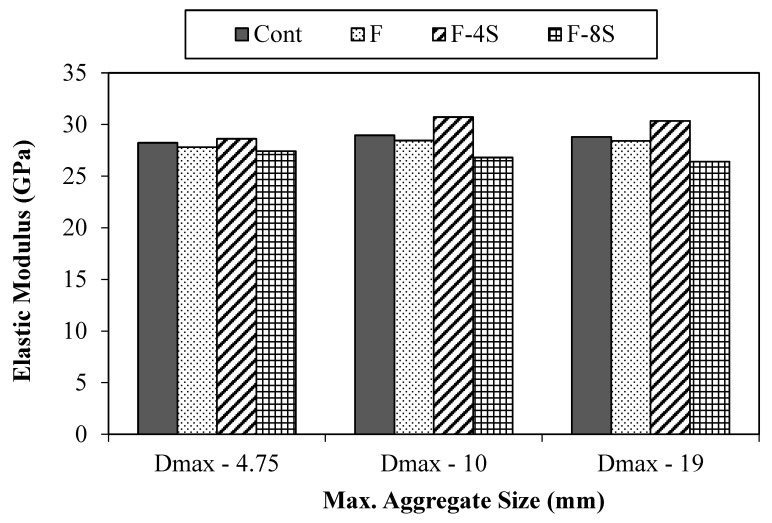
Elastic modulus results.

Further, the elastic modulus of all concrete mixes was also computed experimentally, and results are shown in Figure 9. The value of MOE for all concrete mixes presented here is an average of three samples. It can be noticed that, in comparison of the control mix, the variation in the MOE values due to the addition of fibers and SBR latex is similar to that of compressive strength, i.e., F-4S mix has the highest values of MOE, as compared to their respective control mix, while F-8S has the lowest.

#### Split Cylinder Tensile Strength (f_t_)

**Figure 10:**
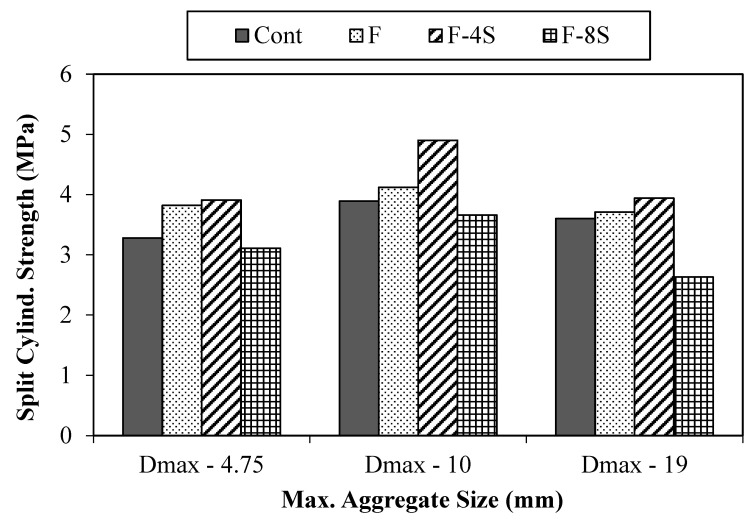
Split cylinder tensile strength results.

For each concrete mix, two specimens were tested, and average results are presented in Figure 10. From the results, it was observed that the addition of 0.5% fibers in concrete improves the indirect tensile strength due to the bridging action of fibers. When 4% SBR latex was added to fibrous concrete, the indirect tensile strength of the resulting concrete was enhanced. The maximum value of indirect tensile strength was observed for the case of the 10-F-4S mix, which was almost 26% higher (4.9 MPa) than the value (3.89 MPa) attained by the corresponding control mix. Whereas, it was 9.45% higher for the 19-F-4S mix. The improvement in the tensile strength is mainly due to the enhanced density of concrete because of the 4% SBR latex and the bridging action of fibers.

On the contrary, the addition of 8% SBR latex showed a negative effect on the indirect tensile strength similar to that of compressive strength. The maximum drop of 27% was exhibited by a concrete mix made using the maximum aggregate size of 19 mm, 0.5% fibers, and 8% SBR latex (19-F-8S) when compared to the corresponding control mix. The drop in the indirect tensile strength of fibrous concrete mix in the case of 8% SBR latex is attributed to the excessive formation of polymerization structure due to the overdosage of SBR latex.

### 3.2. Impact Resistance

#### 3.2.1. Charpy Impact Test

**Figure 11:**
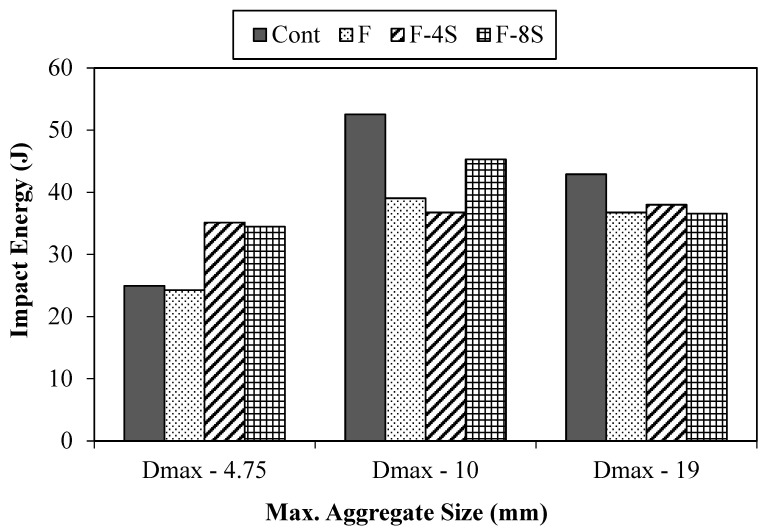
Results from the Charpy impact test.

The Charpy impact tests were performed according to the ASTM standard A370 [26]. Three specimens were tested for each concrete mix. From impact test results, energy was calculated for each concrete mix and the values are provided graphically presented in Figure 11. Here, it is obvious that when 0.5% fibers were added, the absorbed energy under impact loading reduced because of the smaller modulus value of elasticity of polypropylene fibers. However, the addition of 4% SBR latex in fibrous concrete resulted in an improved value of impact energy in comparison to the control mix. Similar to the mechanical properties, the impact resistance of fibrous concrete decreased when 8% the SBR latex was added to concrete due to already mentioned possible reasons.

The results revealed that all concrete mixes with a maximum aggregate size of 19 mm (D_max_-19) showed smaller energy absorption values as compared to the concrete mixes with maximum aggregate sizes of 4.75 mm and 10 mm. The reasons for this smaller energy absorption impact for concrete mixes with D_max_-19 may be linked to the ratio of the size of a specimen prepared for this test and the maximum particle size of aggregate used.

The maximum improvement of 90% was achieved by 4.75-F-4S concrete mix and its receptive compared to the control mix. The improvement in impact energy due to the addition of SBR latex is mainly due to the formation of polymer structure, which results in an enhancement of concrete density and a stronger microstructure. Furthermore, the larger fiber length over the aggregates’ size ratio also positively contributes to energy absorption capacity. However, when 8% SBR latex was added, the impact energy of the resulting concrete mix reduced, and it was found to be even smaller than the impact energy of the control mix. The maximum drop of 17% (37 vs. 43) was exhibited by (19 + F + 8S) when compared to the corresponding control mix.

**Figure 12:**
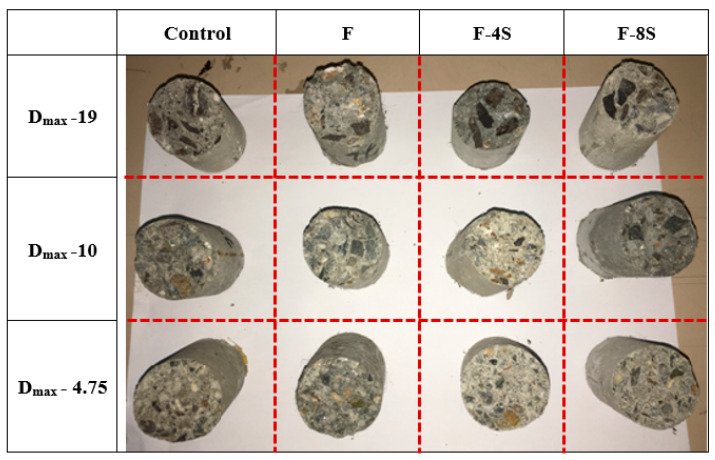
Failed samples of the Charpy impact test (sample size = 40*ϕ* × 100 mm).

In addition, due to larger aggregate sizes, the hammer could directly stroke over the aggregate particles and energy did not distribute to the adjoining aggregates. This quite evident from the failure mode obtained from the Charpy impact test, as shown in Figure 12. Where a smoother surface was obtained for smaller aggregates, high-energy absorption characteristics were indicated. However, for larger aggregates (i.e., D_max_-19), the fracture concentered around the aggregates.

#### 3.2.2. Drop Weight Test on 100 mm × 63.5 mm Concrete Disc

**Figure 13:**
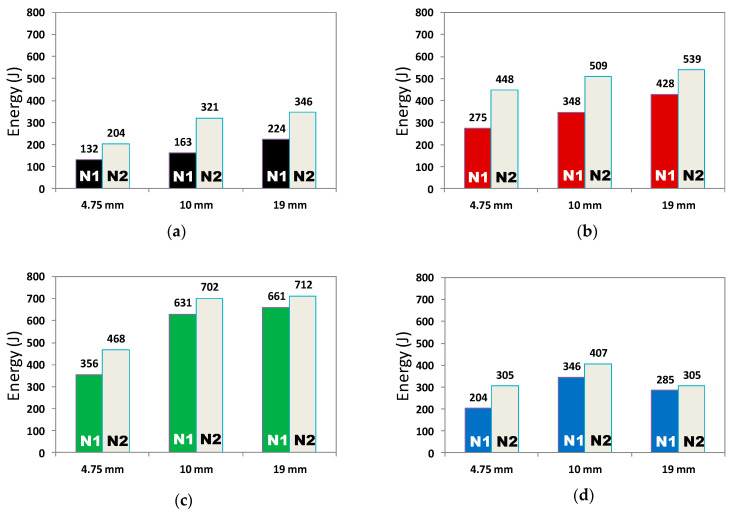
Results of drop weight test on disc specimen (**a**) control specimens, (**b**) with (F) 0.5% fibers, (**c**) with (F-4S) 0.5% fibers and 4% SBR, and (**d**) with (F-8S) 0.5% fibers and 8% SBR.

**Figure 14:**
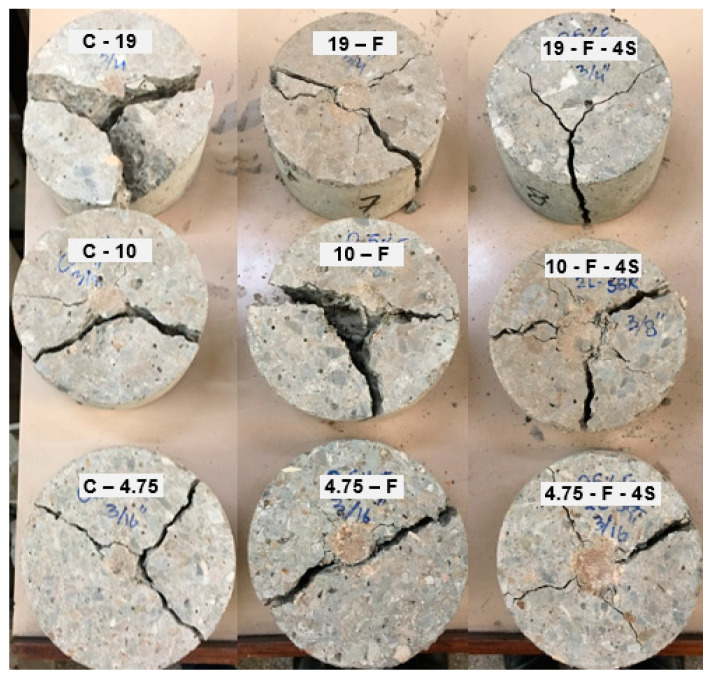
Failure pattern of disc sample (Y-shape).

**Figure 15:**
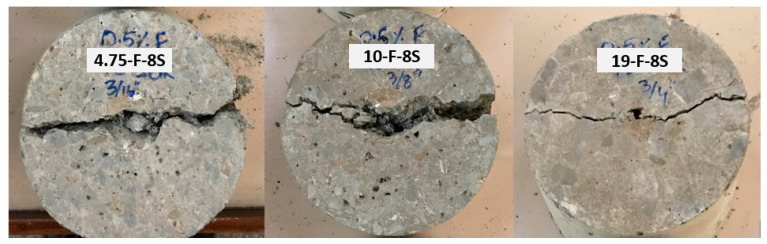
Failure pattern of the disc sample (center split pattern).

Drop weight tests were performed according to ACI 544 [19]. Three specimens were tested for each concrete mix and the average values of impact energy, corresponding to the initial and final crack, was determined, as shown in Figure 13. The initial crack was the stage when the first crack appeared on the concrete surface and a pin needle could be inserted in the crack, while the final crack was the stage when the sample was disintegrated into parts, as shown in Figure 14 and Figure 15. It is pertinent to note that the trend of increase or decrease in the energy absorption attained by concrete mixes in this test was similar to that observed in the compression test.

When fibers were added to concrete, the impact resistance increased because the polypropylene fibers delayed the crack propagation. A significant increase in the impact resistance of concrete can be seen when 4% SBR latex was added. On the contrary, when 8% SBR latex was added to concrete, the impact resistance was decreased. This reduction in impact resistance of concrete may be due to excess formation of the C-S-H structure, which made the concrete brittle. Concrete mixes with 0.5% fibers and 4% SBR latex (F-4S Figure 13a) attained the maximum improvement of 2.29 times (468 J vs. 204 J in N2 for 10-F-4S) when compared to the control mix, as evident in Figure 13c. The improvement in impact energy due to the addition of SBR latex is mainly due to the formation of polymer structure, which results in an enhancement of concrete density and a stronger microstructure. Furthermore, the presence of fibers also positively contributes to energy absorption capacity. However, when 8% SBR latex was added, the impact energy of the resulting concrete mix reduced. The maximum drop of 12% (386 J vs. 305 J in N2) was exhibited by concrete mix 19-F-8S (see Figure 13d) when compared to the corresponding control mix.

Another trend observed in drop weight testing was the increase in impact resistance with the increase in aggregate size. This trend can be observed in all the concrete groups; control mix, i.e., Cont., F, F-4S, and F-8S (concrete with 0.5% fibers, concrete with 4% SBR and fibers, and concrete with 8% SBR and fibers). The maximum improvement of 57% (204 J to 321 J in N2) was achieved by the control concrete mix when the aggregate size increased from 4.75 mm to 10 mm. Furthermore, the same increase in impact resistance energy was observed in concrete mixes F, F-4S, and F-8S, when the aggregate size increased from 4.75 mm to 10 mm. However, when the maximum aggregate size increased from 10 mm to 19 mm, the increase in impact energy was only about 7% to 8%, due to the smaller fiber size as compared to aggregates

Two distinct types of failure pattern of disc sample, namely Y-shape and center split failure, as shown in Figure 14 and Figure 15, were observed in the drop weight samples. The Y-shape failure pattern was observed for control samples, samples with 0.5% fiber, and samples with 0.5% fiber + 4% SBR latex (F-4S), which indicate the ductile behavior of specimens and high-energy absorption characteristics. On the other hand, center split failure was observed for concrete samples with 0.5% fiber and 8% SBR latex (F-8S). This indicates that when the dosage of SBR latex increased from 4% to 8%, it made the concrete brittle, which is not desired under impact loading.

#### 3.2.3. Drop Weight Test on U-Shape Specimen

**Figure 16:**
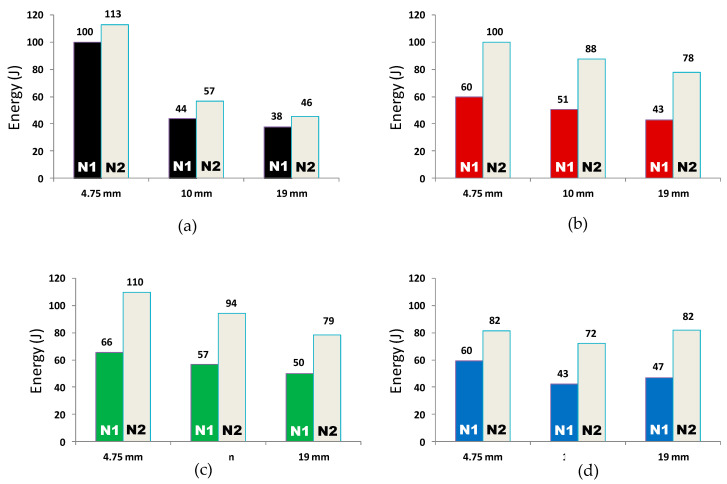
Results of drop weight test on U-shape specimens (**a**) control specimens, (**b**) with (F) 0.5% fibers, (**c**) with (F-4S) 0.5% fibers and 4% SBR, (**d**) with (F-8S) 0.5% fibers and 8% SBR.

The tests on U-shape concrete specimens were also performed according to ACI 544 [19] and guidelines given by Zhu et al. [20]. Three specimens were tested for each mix and average values of impact energy are reported in Figure 16. It is important to mention here that the difference in the trend of impact energy values obtained for concrete disc and U-shape specimens of all concrete mixes made for this study is mainly attributed to different loading conditions in both cases. In the former case, it is compression, while in the latter case, loading is flexure. The impact resistance of concrete mix with a maximum aggregate size of 4.75 mm decreased when fibers were added. A significant increase in the impact resistance was obtained when 4% SBR latex was added. Nevertheless, when 8% SBR latex was added, the impact resistance slightly reduced.

**Figure 17:**
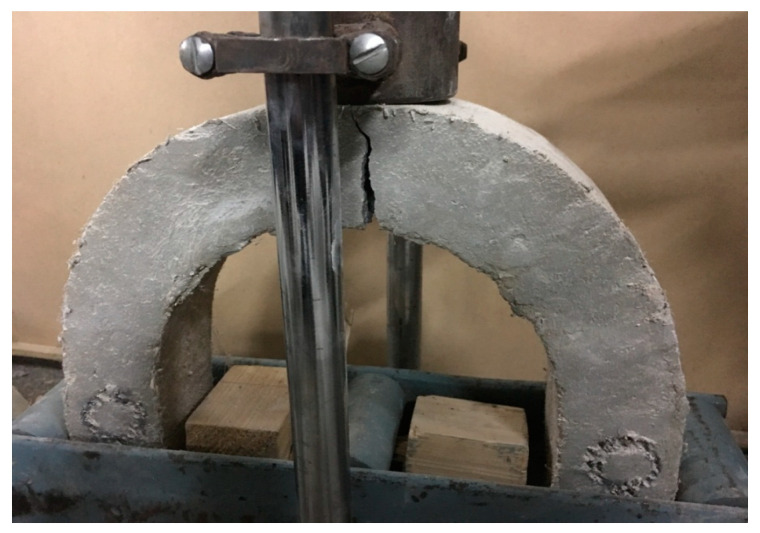
Final failure crack in U-shape specimen under the drop weight test.

From the results presented in Figure 16b, it can be observed that when fibers were added in 10 mm and 19 mm aggregate concretes, the impact resistance of the concrete increased due to better anchorage and bridging action. The maximum improvement of 72% (46 J to 79 J for 19-F-4S) was achieved by concrete mix with 0.5% fibers and 4% SBR latex as compared to the control mix. This increment in energy absorption is also in line with test results conducted on disc specimens. The typical failure of U-shape specimens subjected to impact loading is shown in Figure 17, where it is clear that the loading mode was flexure instead of compression, which resulted in the gradual propagation of a single localized crack from the bottom to the top.

## 4. Conclusions

Based upon the experimental results the following conclusions can be drawn.

(1) The addition of polypropylene fibers has an insignificant effect on mechanical strength. However, with the addition of 4% SBR latex, strength values (compressive and tensile) increased. Further, the addition of 8% SBR latex had a negative impact on the mechanical properties of the polypropylene fiber-reinforced concrete.
(2) The impact resistance of polypropylene fiber-reinforced concrete determined through the drop weight test on disc specimen was enhanced by the addition of 4% SBR latex. This enhancement was 1.7 to 2.9 times up to the initial crack, while it was 1.05 to 1.30 times the value of the control concrete mix at final failure.
(3) For the concrete containing 4% SBR latex, after the initial cracking of disc specimen under impact loading, the presence of polypropylene fibers caused a 32% increase in energy absorption capacity up to final failure which was 55% in the case of the control mix.
(4) With the increase in maximum aggregate size from 4.75 mm to 19 mm, the compressive strength and impact resistance of concrete (obtained through drop weight test on disc specimen) increased by 20% and 75%, respectively.

The results obtained for impact resistance of concrete through the drop weight test on the disc specimen and the U-shape specimen cannot be compared, as the mode of failure is different in both cases. There is compression in the case of the disc specimen and flexure in the case of the U-shape specimen. Furthermore, in a U-shape sample, the impact force is more localized as compared to the regular disc.

(5) Experimental observations showed that the Charpy test is not reliable for large-size aggregate concrete with respect to the sample size used in this study.

## Figures and Tables

**Table 1:** Aggregate properties.

	Aggregate Type	
	Sargodha Crush	Lawrencepur Sand
Bulk density (kg/m^3^)	1630	1840
Fineness modulus	-	2.3
Specific gravity	2.72	2.60
Voids (%)	42.8	25.6
Impact value (%)	16.50	-
Crushing value (%)	29.80	-

**Table 2:** Characteristics of SBR latex.

Property	Value
Density	1 kg/L
Solids content (by weight)	48%
pH	10–11

**Table 3:** Characteristics of polypropylene fiber.

Property	Value
Density	0.9 ± 0.01 kg/L
Fiber length	12 mm
Fiber diameter	Approx. 15–30 micron
Absorption	Nil
Tensile strength	300–450 MPa
Elongation at break	>15%
Softening point	160 °C
Specific surface area	Approx. 200 m^2^/kg
Thermal conductivity	Low

**Table 4:** Details of the mix design.

Sr. No.	MIX ID	PP Fibers ^†^	SBR Latex ^†^	Cement kg/m^3^	Coarse Aggregate kg/m^3^	Fine Aggregate kg/m^3^	Water kg/m^3^	W/C	Max. Coarse Aggregate Size, mm
1	C-4.75	--	--	410	1230	615	164	0.4	4.75 mm
2	4.75-F	0.5	--	410	1230	615	164	0.4	
3	4.75-F-4S		4	410	1230	615	164	0.4	
4	4.75-F-8S		8	410	1230	615	164	0.4	
5	C-10	--	--	405	1220	610	163	0.4	10 mm
6	10-F	0.5	--	405	1220	610	163	0.4	
7	10-F-4S		4	405	1220	610	163	0.4	
8	10-F-8S		8	405	1220	610	163	0.4	
9	C-19	--	--	400	1190	595	160	0.4	19 mm
10	19-F	0.5	--	400	1190	595	160	0.4	
11	19-F-4S		4	400	1190	595	160	0.4	
12	19-F-8S		8	400	1190	595	160	0.4	

^†^ Percentage by cement weight.

**Table 5:** Test specimen details.

Specimen	Specimen Size	No. of Specimens		Tests to Be Performed
		Per Test	Total	
Cylinder	150*ϕ* × 300	2	24	Compressive strength
Cylinder	150*ϕ* × 300	2	24	Split cylinder strength
Cylinder	150*ϕ* × 300	2	24	Modulus of elasticity
Cylinder	40*ϕ* × 100	4	48	Charpy impact yest
Cylinder	100*ϕ* × 63.5	3	36	Drop weight impact test
U-Shape	See Figure 2	3	36	Drop weight impact test

## Data Availability

The data presented in this study are available upon request from the corresponding author. The data are not publicly available due to privacy.
